# High-Resolution Mapping of Homologous Recombination Events in *rad3* Hyper-Recombination Mutants in Yeast

**DOI:** 10.1371/journal.pgen.1005938

**Published:** 2016-03-11

**Authors:** Sabrina L. Andersen, Aimee Zhang, Margaret Dominska, María Moriel-Carretero, Emilia Herrera-Moyano, Andrés Aguilera, Thomas D. Petes

**Affiliations:** 1 Department of Molecular Genetics and Microbiology, Duke University, Durham, North Carolina, United States of America; 2 Department of Molecular Biology, Centro Andaluz de Biología Molecular y Medicina Regenerativa CABIMER-Universidad de Sevilla, Seville, Spain; NYU Langone Medical Center, UNITED STATES

## Abstract

The *Saccharomyces cerevisae RAD3* gene is the homolog of human *XPD*, an essential gene encoding a DNA helicase of the TFIIH complex involved in both nucleotide excision repair (NER) and transcription. Some mutant alleles of *RAD3* (*rad3-101* and *rad3-102*) have partial defects in DNA repair and a strong hyper-recombination (hyper-Rec) phenotype. Previous studies showed that the hyper-Rec phenotype associated with *rad3-101* and *rad3-102* can be explained as a consequence of persistent single-stranded DNA gaps that are converted to recombinogenic double-strand breaks (DSBs) by replication. The systems previously used to characterize the hyper-Rec phenotype of *rad3* strains do not detect the reciprocal products of mitotic recombination. We have further characterized these events using a system in which the reciprocal products of mitotic recombination are recovered. Both *rad3-101* and *rad3-102* elevate the frequency of reciprocal crossovers about 100-fold. Mapping of these events shows that three-quarters of these crossovers reflect DSBs formed at the same positions in both sister chromatids (double sister-chromatid breaks, DSCBs). The remainder reflects DSBs formed in single chromatids (single chromatid breaks, SCBs). The ratio of DSCBs to SCBs is similar to that observed for spontaneous recombination events in wild-type cells. We mapped 216 unselected genomic alterations throughout the genome including crossovers, gene conversions, deletions, and duplications. We found a significant association between the location of these recombination events and regions with elevated gamma-H2AX. In addition, there was a hotspot for deletions and duplications at the *IMA2* and *HXT11* genes near the left end of chromosome XV. A comparison of these data with our previous analysis of spontaneous mitotic recombination events suggests that a sub-set of spontaneous events in wild-type cells may be initiated by incomplete NER reactions, and that DSCBs, which cannot be repaired by sister-chromatid recombination, are a major source of mitotic recombination between homologous chromosomes.

## Author Summary

The two members of each pair of homologous chromosomes in diploid eukaryotes are usually heterozygous for many single-nucleotide polymorphisms (SNPs). Mitotic recombination between homologous chromosomes can lead to loss of heterozygosity of these SNPs. Although mitotic recombination can be beneficial as a source of genetic diversity, loss of wild-type alleles of tumor suppressor genes in heterozygous individuals is an important contributor to carcinogenesis. In the current study, we map recombination events in yeast strains that have mutations in *RAD3* that result in a strong hyper-recombination phenotype. *RAD3* (a homolog of the essential human *XPD* gene) has roles in both transcription and nucleotide excision repair. We find that the *rad3* mutants have a 100-fold elevated rate of crossovers throughout the yeast genome, and these events are non-randomly associated with regions of the yeast genome that are fragile, even in wild-type cells. We also show that most of the recombination events between homologous chromosomes are a consequence of the repair of two sister chromatids that are broken at the same position, similar to the types of events that occur in wild-type cells.

## Introduction

Rad3, the *Saccharomyces cerevisiae* homolog of human XPD, is a 5’ to 3’ DNA helicase that is a subunit of the TFIIH RNA polymerase II initiation factor complex [1,2]. As part of the TFIIH complex, it has roles in transcription initiation and nucleotide excision repair (NER) [3]. In transcription, XPD acts as a structural subunit that links the TFIIH core subunit and the CDK-activating kinase (CAK) subcomplex [4,5]. During NER, Rad3/XPD unwinds the DNA containing a UV-induced lesion, producing double-stranded to single-stranded transitions that are substrates for the endnucleases Rad1-10/XPF-ERCC1 and Rad2/XPG [6]. These nucleases make cuts flanking the lesion, allowing its removal. The resulting single-stranded gap is then filled by DNA polymerase, followed by ligation to complete the repair.

Although the Rad3p is required for viability because of its role in transcription, hypomorphic alleles with a hyper-Rec phenotype have been isolated [7]. These mutations were originally called *rem* (*re*combination and *m*utation). After the demonstration that the *rem1* mutations were allelic with *RAD3*, the *rem1-1* and *rem1-2* alleles were re-named *rad3-101* and *rad3-102* [8]. The Rad3-101 and Rad3-102 proteins are proficient for the transcriptional function of Rad3, but partially deficient in the NER function [9]. These mutants are resistant or only moderately sensitive to UV radiation, respectively [10]. In *rad3-102* strains, the mutant helicase is still capable of separating DNA strands to allow cleavage of the damaged strand. The NER defect occurs post-incision, and it has been postulated that TFIIH remains at the single-stranded intermediate, inhibiting DNA synthesis of the gapped NER intermediate and its subsequent ligation [9]. Replication of the DNA molecule with a single-stranded gap would result in a recombinogenic DSB.

The *rad3-102* mutation alters an amino acid within the ATP-binding groove, and may produce the mutant phenotype by inhibiting ATP hydrolysis, resulting in increased single-stranded DNA binding [9,10]. The *rad3-101* mutation also lies within the ATP hydrolysis groove, and *rad3-101* mutants have even less UV sensitivity than *rad3-102* strains [10]. However, whereas G_1_-synchronized *rad3-102* cells irradiated with 40 J/m^2^ UV-C can progress through S phase, UV-treated *rad3-101* cells are blocked at S phase [10]. FACS analysis indicates that upon UV-treatment *rad3-101* mutants initiate the S-phase but replication is slowed; *rad3-102* cells are only impaired at a higher dose of 100 J/m^2^ [10]. Thus, it is clear that, although the two mutations result in some shared phenotypes, they are not fully equivalent, perhaps differing by their effects on TFIIH single-stranded DNA binding affinity. As described below, in our assay, the hyper-Rec phenotypes of *rad3-101* and *rad3-102* strains are indistinguishable.

In previous studies [7–9], *rad3-101* and *rad3-102* mutants were found to have elevated levels of heteroallelic recombination, and the *rad3-102* mutant had elevated sister-chromatid exchange (SCE). To further define the recombinogenic effects of these mutations, we utilized a color-sectoring assay (described below) that enables us to capture both halves of a reciprocal recombination event [11] in combination with high-resolution mapping using oligonucleotide single-nucleotide polymorphism (SNP) microarrays [12]. We determined that both *rad3-101* and *rad3-102* have 100-fold increased rates of reciprocal crossover (CO) events compared to wild-type, while the rates observed in *rad3-101* and *rad3-102* strains were not significantly different. Also, intriguingly, for both strains, most COs were associated with patterns of gene conversion that indicate the repair of two sister chromatids broken at the same site (double sister-chromatid breaks, DSCBs). It should be emphasized that this finding could not have been made without the colony-sectoring assay that allows detection of all recombinant products.

In addition to elevated levels of crossovers, we observed increased frequencies of other types of genomic rearrangements including gene conversions (GCs) unassociated with crossovers, deletions, duplications, and break-induced replication events. Although these events occurred throughout the genome with no strong hotspots, there is a significant association between the recombination breakpoints and regions with elevated frequencies of the damage associated histone modification gamma-H2AX [13]. We also observed that the sub-telomeric region on the left arm of chromosome XV has a high level of deletions and duplications in the *rad3* hyper-Rec strains.

Although there were quantitative differences in the patterns of recombination observed in the *rad3* versus wild-type strains, certain features (such as the fraction of events initiated by double-chromatid breaks compared to single-chromatid breaks) were very similar. One interpretation of this similarity is that a sub-set of spontaneous mitotic recombination events is initiated by an incomplete NER reaction.

## Results

### Rate of reciprocal crossovers measured using a sectoring assay

**Fig 1:**
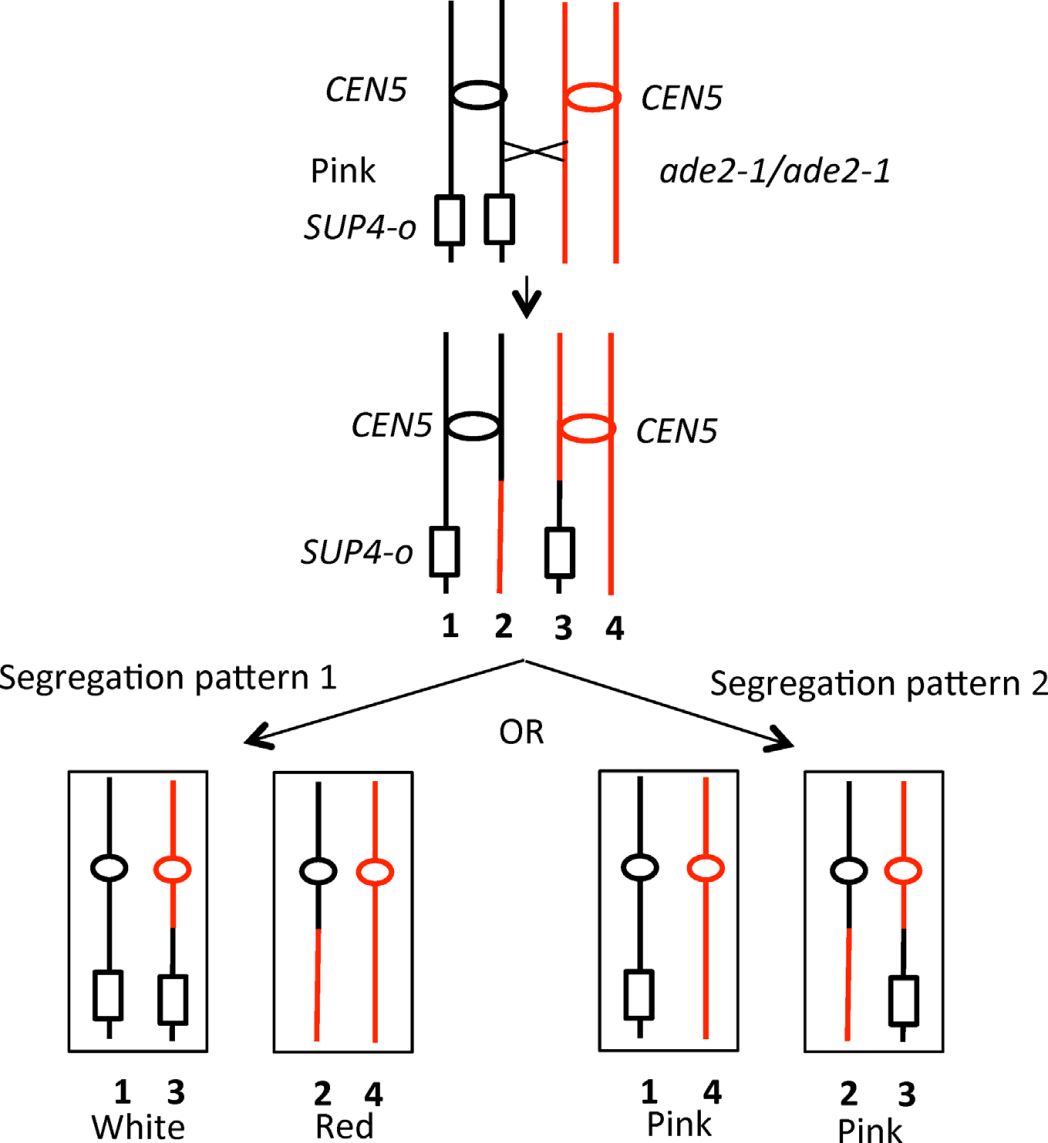
Loss of heterozygosity (LOH) resulting from a crossover on the left arm of chromosome V. Diploid strains that are homozygous for the *ade2-1* mutation and have one copy of *SUP4-o* near the left end of V form pink colonies. A crossover between *SUP4-o* and *CEN5* (shown as an oval), followed by segregation of one recombinant and one non-recombinant chromosome into daughter cells (Segregation pattern 1), will result in a red/white sectored colony. If the two recombinant chromosomes segregate into one daughter cell, and the two non-recombinant chromosomes segregate into the other (Segregation pattern 2), no sectored colony is observed.

The system that we used to select crossovers on the left arm of chromosome V has been described previously [11,14]. The diploid strain was constructed by mating haploid strains derived from the W303-1A [15] and YJM789 [16] genetic backgrounds. In our previously described wild-type diploid PSL101, one copy of chromosome V has the *can1-100* gene (an ochre mutation) located about 32 kb from the left telomere [14]. On the other copy of chromosome V, the *can1-100* gene was replaced with the *SUP4-o* tRNA gene, encoding an ochre suppressor. The strain is also homozygous for the *ade2-1* ochre mutation on chromosome XV. When this mutation is unsuppressed, the diploid forms a red colony. The PSL101 diploid forms pink colonies because the *SUP4-o* gene partially suppresses the *ade2-1* mutation. As shown in Fig 1, if a mitotic crossover occurs on the left arm of chromosome V and the two recombinant chromosomes segregate into different daughter cells, it results in two daughter cells with reciprocal patterns of loss of heterozygosity (LOH), producing a red/white sectored colony (Segregation pattern 1, left side of Fig 1). If the two recombinant chromosomes segregate into the same daughter cell (Segregation pattern 2, right side of Fig 1), an unsectored pink colony will be formed, identical phenotypically to a colony derived from a parental cell without a recombination event. Since these two segregation patterns are equally frequent [17], the rate of crossing over on the left arm of chromosome V can be determined by doubling the frequency of red/white sectored colonies.

The rate of sectored colonies in the wild-type strain was previously determined to be 3.3 x 10^−6^/division [14]. In isogenic diploids homozygous for *rad3-101* (SLA64.1) and *rad3-102* (MD555/MD556), the rates of sectors were elevated to 2.5x10^-4^/division and 5.0x10^-4^/division, respectively). The approximately 100-fold elevations in the rates of crossovers in the two strains were not significantly different from each other.

### Classes of recombination events in *rad3-101* and *rad3-102* strains

As shown in Fig 1, mitotic crossovers result in loss of heterozygosity (LOH) for markers located centromere-distal to the recombination event. To map patterns of LOH in each sector of the sectored colonies, we used oligonucleotide-containing microarrays [12]. The diploids employed in our study were derived by crosses of two sequence-diverged haploid strains: YJM789 and W303-1A. The sequences of these strains differ by about 55,000 single-nucleotide polymorphisms (SNPs) [14]. We designed two types of SNP microarrays that allowed us to determine whether strains were homozygous or heterozygous for SNPs located on chromosome V (V-specific SNP array) or throughout the genome (whole-genome SNP arrays). For the V-specific array, we examined about 750 SNPs distributed throughout the 577 kb chromosome. Each SNP was represented on the array by four 25-base oligonucleotides, two identical to the Watson and Crick sequence of the W303-1A-derived SNP and two identical to the YJM789-derived SNP. The sequences used for the oligonucleotides for the V-specific array are listed in S1 Table. For the whole genome array, we used about 53,000 oligonucleotides (13,000 SNPs), allowing us to examine LOH events throughout the genome at approximately one kb resolution [12].

The analysis of LOH with microarrays has been described in detail by St. Charles *et al*. [12]. In brief, genomic DNA is isolated from cells derived from either the red or white part of the sector. This DNA is labeled with Cy5-dUTP. DNA from the control heterozygous parental diploid is labeled with Cy3-dUTP. The samples are mixed and hybridized to the SNP-microarrays (details in Materials and Methods). By determining the ratio of hybridization to the Cy5- and Cy3-labeled probes for each SNP, we could determine whether the sample was heterozygous, homozygous for the YJM789-derived SNP, or homozygous for the W303-1A-derived SNP. This analysis allows us to detect recombination events leading to LOH as well as genomic amplifications and deletions. We examined 33 sectored colonies derived from the *rad3-102* strain, and 10 derived from the *rad3-101* strain. Results from these two strains were not significantly different.

**Fig 2:**
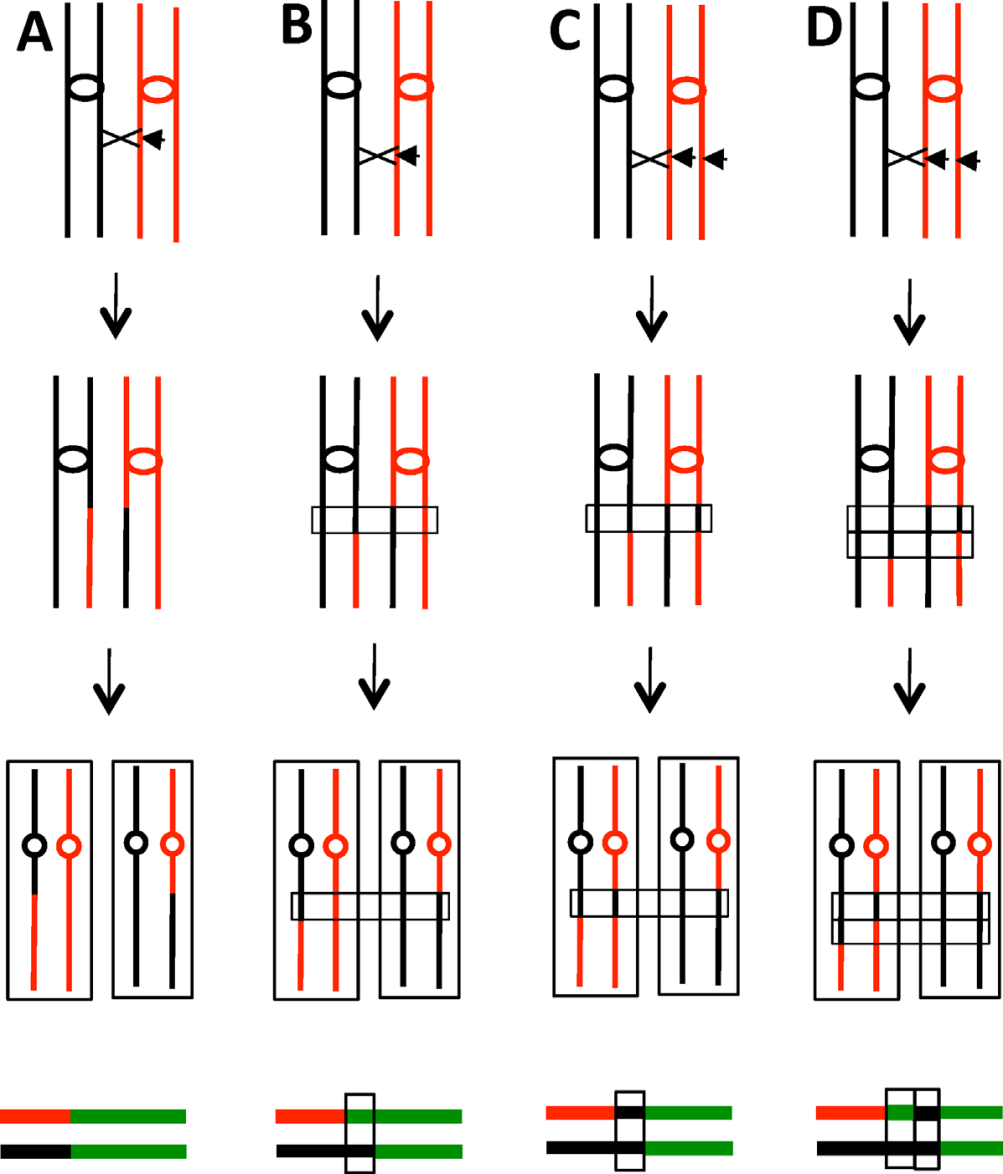
Crossovers and associated gene conversion events in red/white sectored colonies. In the figure, duplicated YJM789- and W303-1A-derived chromatids are shown as black and red lines, respectively. Centromeres are shown as ovals, and crossovers as X's. Short horizontal arrows indicate the site of the recombinogenic DNA lesion. At the bottom of each diagram, we show a pair of lines that depict patterns of LOH. The top and bottom lines summarize LOH patterns in the red and white sectors, respectively, using the following code: green (heterozygous SNPs), red (homozygous W303-1A-derived SNPs), and black (homozygous YJM789-derived SNPs). The boxed regions show gene conversion events as discussed in the text. A. Crossover unassociated with gene conversion. B. Crossover associated with the repair of one broken chromatid, generating a 3:1 conversion event. The region of the conversion is boxed. C. Crossover associated with the repair of two sister chromatids broken at the same position. The repair of one chromatid is associated with a crossover. If the sizes of the conversion tracts are the same for both broken chromatids, a 4:0 conversion event would be produced. D. Crossover associated with the repair of two broken sister chromatids for which the conversion tracts were of different sizes. The net result is a 4:0/3:1 hybrid conversion tract.

Several of the patterns of LOH observed in sectored colonies are shown in Fig 2. In a crossover unassociated with gene conversion, the transition between heterozygous markers and homozygous markers occurs at the same place in the two sectors with the red sector becoming homozygous for W303-1A-derived SNPs distal to the transition point and the YJM789-derived sector becoming homozygous for the YJM789-derived SNPs (Fig 2A). This class of event is relatively rare (1 in 43 sectored colonies), as expected, since both meiotic and mitotic crossovers are usually associated with an adjacent region of DNA in which information is transferred non-reciprocally between the two homologs (gene conversions) [18,19]. In our analysis, conversion events are detected by sectored colonies in which the two transition points are not identical (Fig 2B). In this example, within the boxed region, three of the chromosomes have SNPs derived from YJM789 and one chromosome has SNPs derived from W303-1A (3:1 conversion). Previous studies of gene conversion show that the chromosome that has the DNA lesion, usually assumed to be a double-stranded DNA break (DSB), receives sequences from the intact chromosome [20]. Thus, in the event depicted in Fig 2B, the initiating DSB was on the W303-1A-derived homolog.

**Fig 3:**
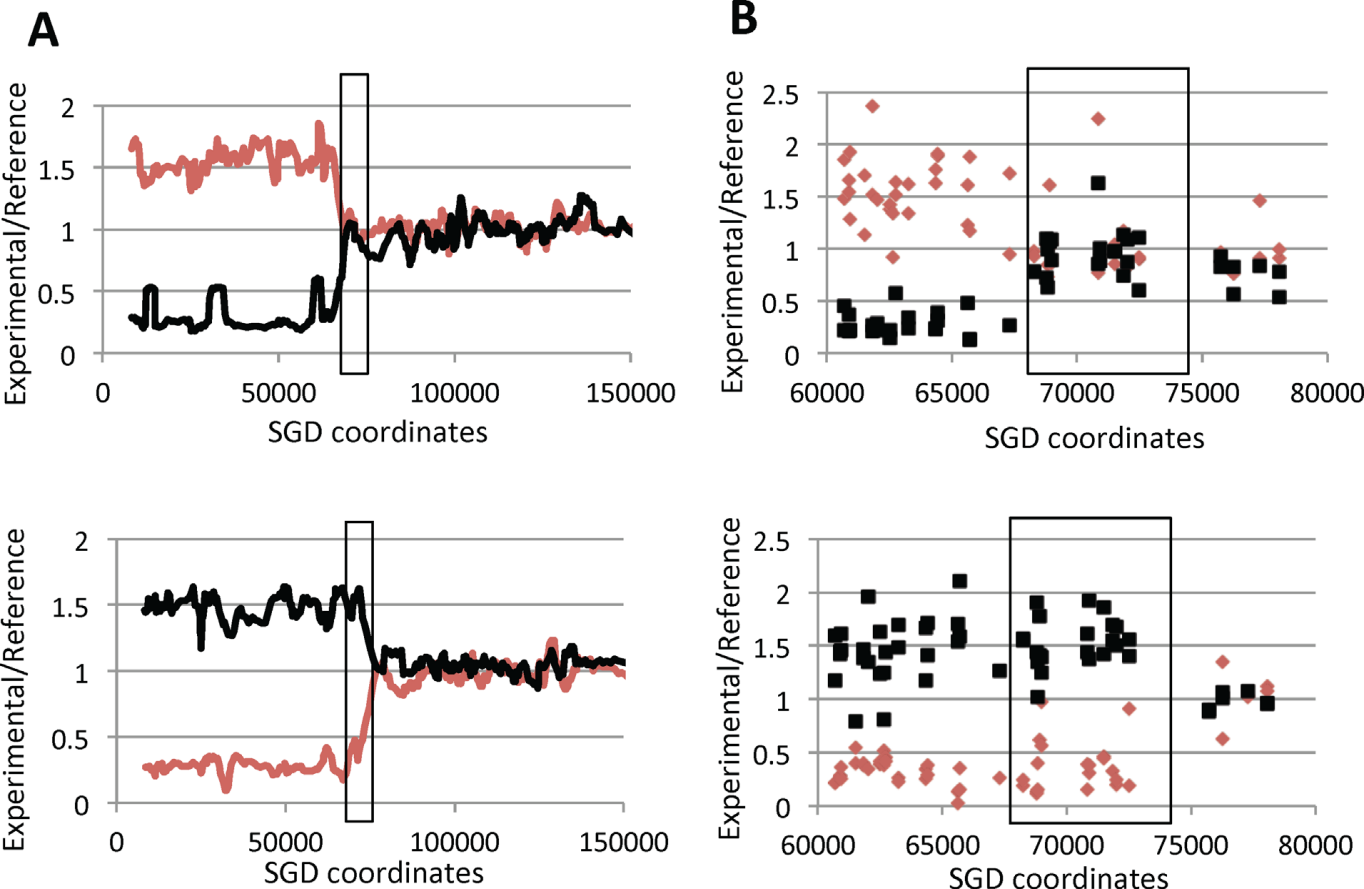
Microarray analysis of a sectored colony (MD555-23.8) generated by a crossover on the left arm of chromosome V associated with a simple 3:1 conversion event. As described in the text, DNAs isolated from the red (top panel) and white (bottom panel) sectors of a colony were hybridized to a microarray containing SNP-specific oligonucleotides. The hybridization levels of each experimental sample are normalized to the levels observed for heterozygous control diploid samples, and the hybridization levels to W303-1A-specific and YJM789-specific oligonucleotides are shown as red and black lines, respectively. The heterozygous *SUP4-o* insertion is located at SGD coordinate 32 kb, and *CEN5* is near coordinate 152 kb. A. Low-resolution depiction of hybridization patterns. Hybridization values were calculated in a moving window of 10 adjacent SNPs. Although the DNA samples have reciprocal patterns of hybridization, the transitions between heterozygous and homozygous SNPs occur at different positions in the two sectors. The boxed region shows the gene conversion event; within this region, three chromosomes have YJM789-specific SNPs and one has W303-1A-specific SNPs. B. High-resolution depiction of hybridization patterns. Small red diamonds and small black boxes represent hybridization levels to individual W303-1A-specific and YJM789-specific oligonucleotides, respectively. The boxed region indicates the conversion event.

In most models of recombination, gene conversion events reflect heteroduplex formation followed by repair of the resulting DNA mismatches [20]. Since heteroduplexes are likely an intermediate in formation of crossovers, most crossovers will have an associated gene conversion event. In our previous analysis [19], we found that about 90% of mitotic crossovers on chromosome IV were associated with conversions (3:1, 4:0, 3:1/4:0 hybrid, or complex events). In the current analysis, five of the 43 crossovers were associated with 3:1 conversion tracts. One example of a 3:1 conversion event as analyzed by SNP microarrays is shown in Fig 3.

We also observed other patterns of conversions associated with crossovers. In Fig 2C, we show a 4:0 conversion event. In the boxed region, all four chromosomes in the two sectors share information derived from the YJM789-derived homolog. Such events reflect the repair of two sister chromatids broken at the same position. We suggested previously that sister chromatids that share breaks at the same position likely reflect the replication of a chromosome broken in G_1_ [14,21]. In Fig 2C, we show crossover-associated repair of one DSB with the repair of the other DSB unassociated with a crossover. If the repair of both DSBs on the red chromatids are associated with crossovers involving the black chromatids, no LOH will be observed. In addition to 4:0 events, we frequently observed conversion tracts that had both 4:0 and 3:1 regions (Fig 2D). Such tracts are consistent with the repair of two sister chromatids in which the lengths of the conversion tracts associated with the repair are different for the two broken chromosomes. 12 of 43 crossovers were associated with 4:0 or 4:0/3:1 hybrid conversion tracts.

We also show in Fig 2 a schematic depiction of the mapped recombination events. Each sectored colony is represented by a pair of lines with the upper and lower lines representing events in the red and white sectors, respectively. The green, red, and black line segments show regions that are heterozygous, homozygous for W303-1A-derived SNPs, and homozygous for YJM789-derived SNPs, respectively. Similar depictions of all conversion events in sectored colonies are shown in S2 Table. In addition to the 18 relatively simple events described above (Classes S1-S8 in S2 Table), we observed 25 conversions with multiple transitions between heterozygous regions and LOH regions (Classes C1-C25 in S2 Table). Similar complex tracts have been observed in our previous studies [19,22]. As discussed in these studies, such tracts are likely a consequence of “patchy” mismatch repair, strand switching of the invading DNA molecule, and/or formation of long symmetric heteroduplexes.

Of the 18 crossovers that were associated with relatively simple conversion tracts, 5 reflected the repair of a single broken chromatid (single-chromatid breaks; SCBs) and 13 were a consequence of the repair of two broken chromatids (double-sister chromatid breaks; DSCBs). In S2 Table, we also classified most of the complex events as resulting from SCBs or DSCBs. All complex events that had a region of 4:0 tracts were classified as DSCBs. Of the 25 complex events, 18 were DSCBs, 4 were SCBs, and 3 were not classifiable as DSCBs or SCBs. In summary, of the 39 crossovers with diagnostic conversion events, 9 (23%) were SCB-associated (likely reflecting DSBs formed in the S- or G_2_ periods of the cell cycle) and 30 (77%) were DSCB-associated (likely reflected G_1_-initiated DSBs). In wild-type cells, the numbers of events that were SCB- and DSCB-associated were 45 (37%) and 76 (63%), respectively [19]. This distribution of events is not significantly different from that observed in the *rad3* mutant strains (p = 0.12 by Fisher exact test). For both the *rad3-101* and *rad3-102* strains, the majority of conversion events (88% and 73%, respectively) were classified as reflecting DSCB-associated repair.

### Location of recombination events in sectored colonies in *rad3* mutant strains

**Fig 4:**
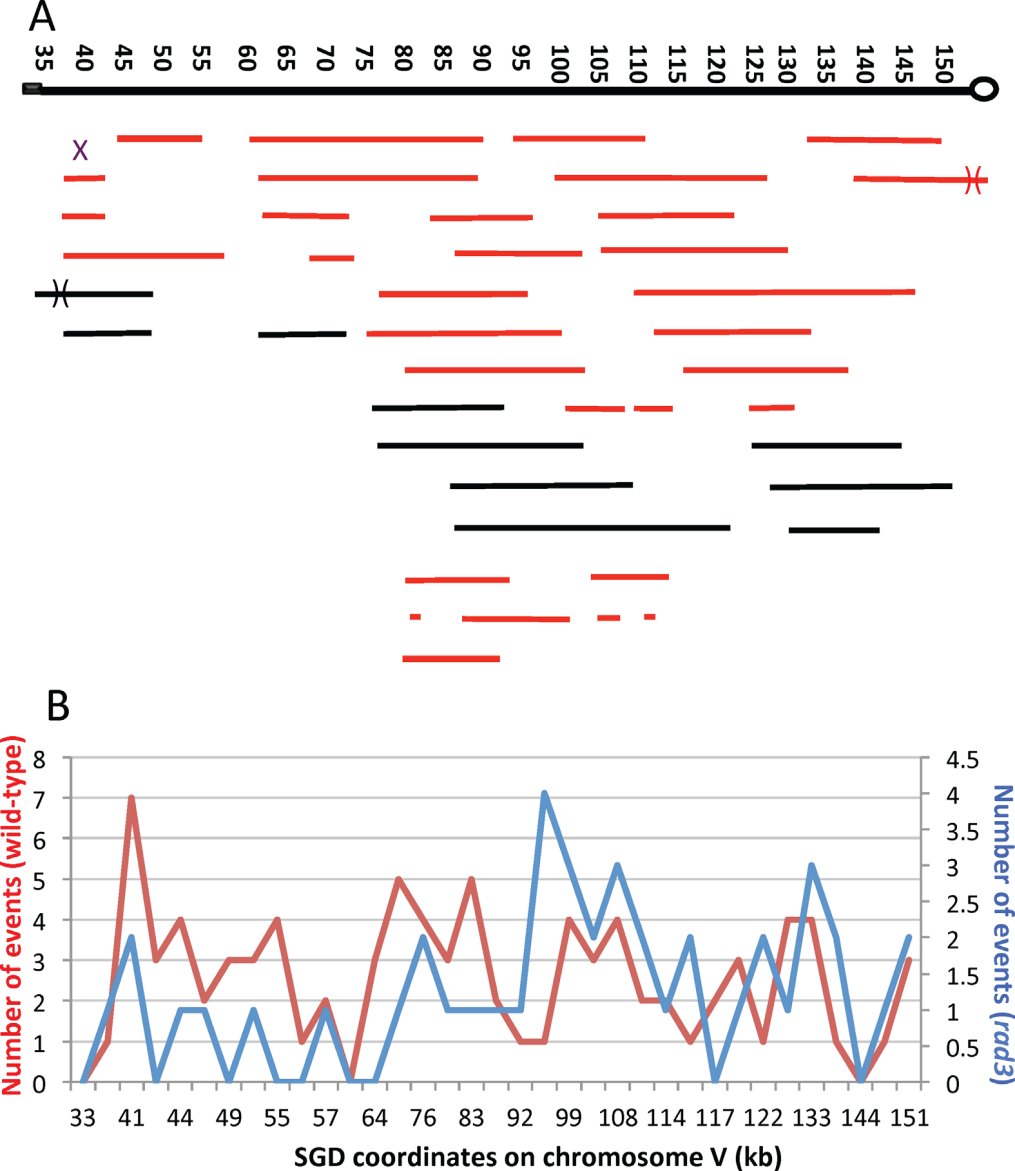
Map locations of crossovers and associated conversions in the *CEN5-SUP4-o* interval of chromosome V. The black rectangle shows the location of *CAN1/SUP4*, and the circle represents *CEN5*. A. Location and lengths of conversion tracts. We mapped 33 events derived from sectored colonies of the *rad3-102* diploids (MD555/MD556; shown as red lines) and 10 events derived from sectored colonies of the *rad3-101* diploid (SLA64.1; shown as black lines). Each continuous line shows a single conversion tract with the length of the line indicating the length of the conversion tract. Numbers at the top of the figure show SGD coordinates in kb, with position 1 representing the left end of chromosome V. The X shows the position of a crossover unassociated with gene conversion. B. Comparison of crossovers in the wild-type (red line) and *rad3* mutant (blue line) strains. In a previous study [14], we mapped crossovers in the wild-type strain using a smaller number of SNPs than employed in the present study. To allow a direct comparison of events in the wild-type and *rad3* strains, we used the same SNPs as employed by Lee *et al*. We determined the mid-points for each conversion event in the *rad3* strains. We then determined which of the SNPs used in the Lee *et al*. study was closest to these midpoints. The numbers of conversion events at each SNP are shown on the Y-axis, and the SGD coordinates for each SNP are on the X-axis. The distances on the X-axis are not proportional to the distances between SNPs.

For each of the sectored colonies, we mapped the positions of all of the LOH transitions associated with the colony. Each transition (shown as small letters in S2 Table) is associated with two Saccharomyces Genome Database (SGD) coordinates, each coordinate indicating the position of the oligonucleotides at the boundaries of the transition. The data with the coordinates of the transitions are in S3 Table. In a sectored colony that results from a crossover without an associated conversion, the two coordinates represent the homozygous and heterozygous SNPs that were closest to the transition. A summary of the location of recombination events of the sectored colonies is shown in Fig 4A. The length of each line indicates the length of the associated conversion tract; for complex tracts, the distances from the beginning of the tract to the end is shown. Red and black colors indicate whether the event was obtained with the diploids homozygous for *rad3-102* and *rad3-101*, respectively.

In Fig 4B, we show the distribution of recombination events on the left arm of chromosome V in the *rad3* strains (blue line in the figure) by assigning each event a single SGD coordinate based on the midpoint of the gene conversion tract associated with the CO or, for simple crossovers lacking a GC tract, the midpoint between the last heterozygous SNP centromere proximal to the CO and the first homozygous SNP. This distribution of events was compared to a previous analysis of recombination events (red line in the figure) in an isogenic wild-type strain [14]. For both the wild-type and *rad3* strains, the events are broadly distributed in the region between the centromere and the *can1-100/SUP4-o* markers with no very strong recombination hotspots. We also divided the interval between the coordinates 35000 and 161000 on the left arm of chromosome V into five bins of approximately 25 kb, and counted the number of events in each bin for the wild-type and *rad3* strains. A comparison of the two sets of data using a 2 x 5 contingency chi-square table indicated a significant (p = 0.04) difference in the distribution of events with the *rad3* strain having more events in the centermost of the five bins. Aside from this difference, the distribution of events in the wild-type and *rad3* strains is quite similar.

### Lengths of conversion tracts associated with crossovers on chromosome V in *rad3* mutant strains

Using the information in S3 Table, we calculated the lengths of gene conversion tracts associated with crossovers. As in previous studies [12], tract length was calculated by averaging the maximum length (the distance between SNPs that were not included in the tract) and the minimum length (the distance between converted SNPs at the ends of the tract). We grouped the tracts based on whether they were DSCB (G_1_) or SCB (G_2_) events, since these two classes have different median tract lengths in wild-type cells [19]. The conversion tracts for the DSCB events in the *rad3-102* and *rad3-101* strains (combined data) had a median length of 18.2 kb (15.6–22.2 kb; 95% confidence limits) compared to a median length of 14.8 kb (11.7–17.5 kb) in the wild-type strain [19]. By a Mann-Whitney test, this difference was statistically significant (p<0.001). The tracts associated with SCB events were also significantly (p = 0.03 by the Mann-Whitney test) longer for the *rad3* strains than for the wild-type strain with median lengths of 7.9 kb (3.2–18.4 kb) and 4.7 kb (2.6–9.5 kb), respectively.

For both the wild-type and *rad3* strains, the conversion events were about two times longer for the DSCB events than the SCB events. This observation is likely a consequence of the mechanism by which DSBs are repaired. During repair, one broken end invades the homologous chromosome [20], and the extent of DNA synthesis from the invading DNA molecule is one of the main determinants of conversion tract length. In SCB events, the tract is propagated toward the centromere or toward the telomere from the point of invasion. In DSB events, since there are two invading strands, the two tracts could be propagated in opposite directions. In our analysis, since we measure the conversion tract as extending from the most centromere-proximal LOH SNP to the most centromere-distal LOH SNP within the sectored colony, this length is the sum of the two conversion tracts for DSCB events which is likely longer than the single tract for the SCB events. As noted previously [12,14], both types of mitotic conversion tracts are considerably longer than meiotic conversion tracts that have a median size of about two kb [18].

One factor that may be related to the longer conversion tracts observed in the *rad3* strains is the *rad3*-associated tracts were significantly more complex than those in the wild-type strain. We consider simple conversion tracts as those whose pattern of LOH can be explained by the canonical double-strand break repair model without invoking branch migration, template switching, or “patchy” repair. Such tracts are shown as Classes S2-S8 in S2 Table; complex tracts are shown as Classes C1-C25. By this definition, 17 of the crossovers in the *rad3* strains were simple and 25 were complex. By the same definition, in the wild-type strain in our previous study, 82 were simple and 39 were complex. This difference is significant (p = 0.002 by Fisher exact test). Possible reasons for this difference will be discussed below.

### Unselected LOH events in *rad3* strains

**Fig 5:**
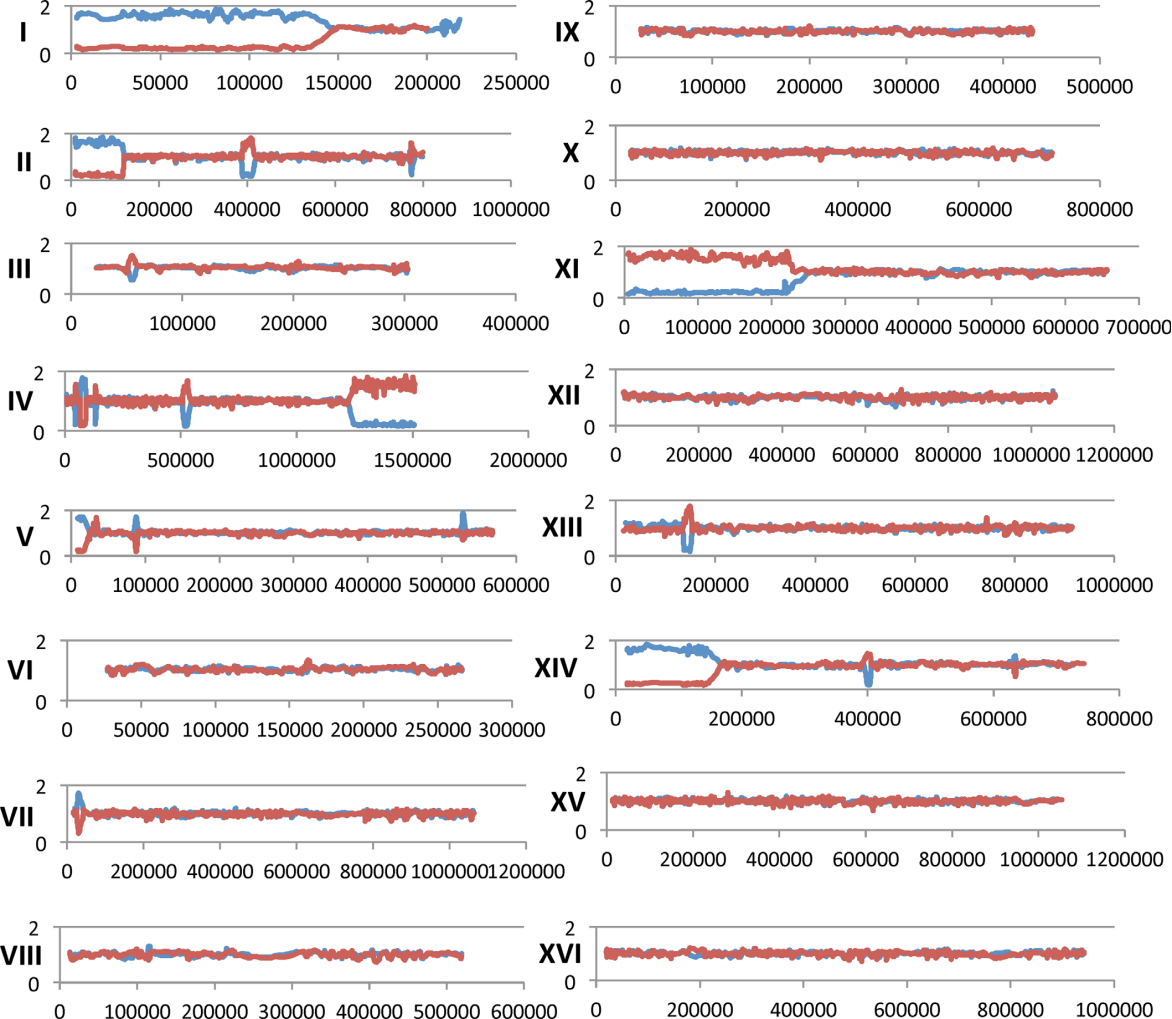
LOH events in one sub-cultured *rad3-102/rad3-102* isolate (MD555-7 B) as determined using SNP microarrays. As shown in this figure, a single sub-cultured isolate frequently has multiple unselected terminal (for example, as in chromosome I) and interstitial (two such events on chromosome II) LOH events. For each figure, the Y-axis reflects the hybridization ratio relative to the heterozygous control strain, and the X-axis contains SGD coordinates. Chromosome numbers are shown in Roman numerals. The red and blue lines indicate hybridization to W303-1A-specific and YJM789-specific SNPs, respectively.

When we analyzed the sectored colonies of the *rad3* mutant strains by whole-genome microarrays, it was evident that these strains had multiple unselected LOH events in addition to the selected crossover on chromosome V. For 33 sectored colonies derived from the *rad3-102* diploids (MD555 and MD556), there was an average of about three unselected LOH events per sectored colony. In order to obtain a larger number of unselected events per isolate, we also did a whole-genome microarray analysis of five sub-cultured samples from each of the *rad3-102* and *rad3-101* diploids. Strains were sub-cultured by ten cycles of allowing single cells to form colonies on solid medium, followed by re-streaking of those colonies to single cells. These sub-cultured isolates had about ten LOH events per isolate. Ten cycles of sub-culturing are equivalent to about 250 cell divisions. In experiments in which an isogenic wild-type diploid was sub-cultured ten times, an average of 0.5 LOH events was observed [23]. These observations confirm the strong (roughly twenty-fold) hyper-Rec phenotype associated with the *rad3-101* and *rad3-102* strains. Previously, Navarro *et al*. [24] observed that the *rad3-102* mutation elevated crossovers in the *CEN5-CAN1* interval on chromosome V by about seven-fold. An example of the LOH events in one sub-cultured isolate is shown in Fig 5.

In order to generate a red/white sectored colony, the crossover on chromosome V must occur in the first cell division after cells are plated on solid medium. Unselected events, however, could occur prior to or subsequent to the selected crossover. For example, if a reciprocal crossover occurred on the left arm of chromosome IV prior to the selected crossover, we could detect an LOH event in one sector (for example, duplicating the YJM789-derived SNPs) without detecting the reciprocal LOH event in the other sector (duplicating W303-1A-derived SNPs). Terminal LOH events (events in which the region of LOH extends to the right or left telomere), therefore, could reflect either a crossover in a previous division or a break-induced replication (BIR) event. BIR events are a consequence of a broken end that invades a homologous template, copying it from the point of invasion to the end; such events are inherently non-reciprocal [20]. Because of this ambiguity, all unselected terminal LOH events are designated “BIR/CO” in S4 Table which shows the SGD coordinates for the transitions of unselected LOH events. The BIR/CO class is about one-third of the approximately 200 LOH events described in this table. The most abundant class of unselected LOH events (representing about two-thirds of the total) is interstitial LOH events. As described previously [23,25,26], such unselected events represent gene conversions. For unselected events, we cannot determine whether the conversion event was associated with a crossover, since events in which the recombinant (crossover) chromosomes co-segregate would yield an interstitial LOH event.

In addition to terminal and interstitial LOH events, about 6% of the events were terminal duplications or deletions (S4 Table). These events involve sub-telomeric regions and there are often sub-telomeric repeated genes located at the transition of the duplication or deletion. For example, the MD555-7 C sub-cultured isolate has a terminal deletion on the left arm of chromosome I with a transition located between coordinates 11599 and 27647. This region contains the *FLO9* gene which has homology to a number of other *FLO* genes located in sub-telomeric regions on other homologs (*FLO1* near the right telomere of I and *FLO5* near the right end of chromosome VIII; SGD). Similar terminal duplications and deletions have also been observed in a yeast study of chromosome rearrangements induced by low levels of DNA polymerase alpha [25]. Such events can be explained as a consequence of a DSB occurring within a sub-telomeric repeat, followed by a BIR event involving a similar repeat on another chromosome. For example, if a DSB occurred in the *FLO9* gene on the left arm of chromosome I and the resulting end was repaired using a *FLO* gene on a non-homologous chromosome, a terminal deletion would be observed on chromosome I. Although this mechanism would be expected to produce a duplication of sub-telomeric sequences from the chromosome that was used as a template in the BIR event, not all of these regions are represented on the microarray because of the redundancy of sequences in the sub-telomeric regions; genomic regions included in the microarray are in Dataset 1 of Song *et al*. [25]. Interestingly, the same region of chromosome I was observed as a terminal deletion in the Song *et al*. study.

**Fig 6:**
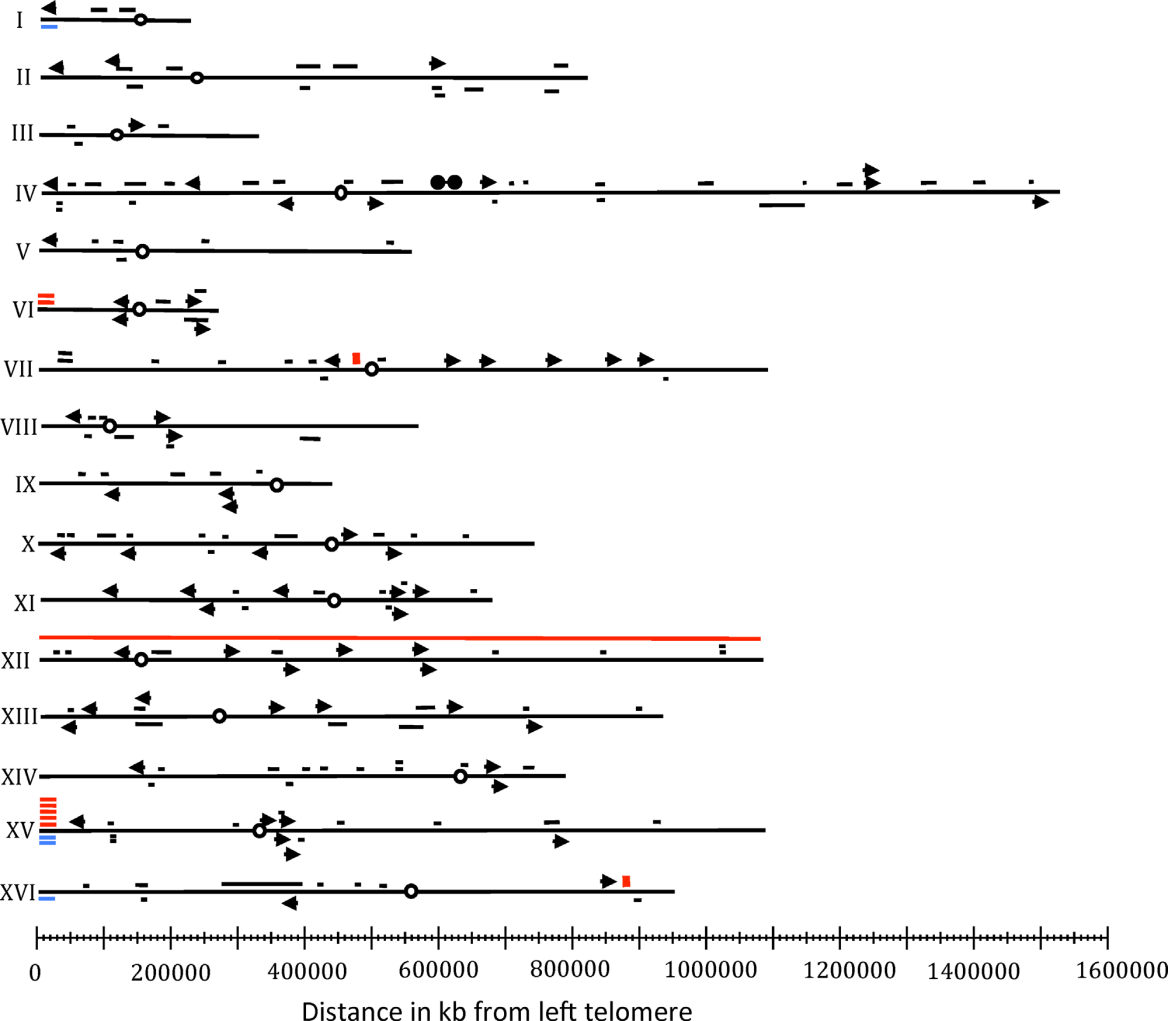
Genome-wide mapping of unselected LOH events in diploids homozygous for the *rad3* hyper-Rec mutations. Data from both sectored colonies (excluding events on the left arm of chromosome V) and sub-cultured isolates are shown. Arrows show terminal LOH events, and short black horizontal lines indicate conversions with the length of the line representing the approximate length of the conversion tract. Red boxes mark the positions of crossovers unassociated with gene conversion. Horizontal red and blue lines indicate terminal duplications and deletions, respectively, and circles represent centromeres. One strain was trisomic for chromosome XII, as indicated by the red line that extends from one telomere to the other.

We observed seven terminal duplication/deletion events involving the left arm of chromosome XV located between coordinates 22345 and 30235 (Fig 6). This region contains two genes (*HXT11* and *IMA2*) that share homology with several genes located in the sub-telomeric regions of other chromosomes. The observed duplications/deletions on XV in the current study likely have two sources. First, as described above, BIR events involving either *HXT11* or *IMA2* interacting with related sequences on non-homologous chromosomes could generate the terminal alterations in isolates MD555 13.8, MD555 23.5, MD556 5.3, and SLA65.1A. Second, in a previous study, we found that LOH events on the left arm of chromosome IX altered the level of hybridization of SNPs located near the left end of XV [23]. Since three of the strains with duplications or deletions on the left end of XV (MD555 15.5, MD555 19.3, MD555 12.1) had LOH events on the left end of IX, these strains likely do not represent true alterations of chromosome XV. Even with the exclusion of these events, there are four strains with terminal duplications/deletions on the left arm of chromosome XV, indicating that this region is “hot” for duplication/deletion events relative to the other chromosome arms. Of 34 terminal duplication/deletions examined in yeast strains with low levels of DNA polymerase alpha, five had a duplication or deletion on XV with the same breakpoint seen in the present study [25]. None of these strains had an alteration on the left arm of IX. In summary, the *HXT* (hexose transporter) and/or the *IMA* genes (isomaltase) are likely hotspots for chromosome rearrangements in yeast strains with the *rad3* mutations or with a partial defect in DNA synthesis.

### Mapping of unselected recombination events

The locations of the unselected recombination events are shown in Fig 6. All chromosomes had at least one event. With the exception of the hotspot for duplications and deletions on chromosome XV, there were no strong recombination hotspots. Gene conversion tracts and regions adjacent to crossovers should contain the sites of recombinogenic DNA lesions [19]. We examined these regions in *rad3* strains to determine whether specific genomic elements (Column 1 of Table 1) were significantly over-represented. Table 1 shows an abbreviated version of this analysis, and a more extended discussion of this type of analysis is provided by St. Charles and Petes [19], Song *et al*. [25], and in the S5 Table. For analysis of terminal LOH events, we used an association window that was 10 kb to each side of the homozygous SNP nearest the recombination breakpoint. For interstitial LOH events, the window included all of the sequences between the heterozygous SNPs flanking the LOH region. We summed these association windows for all of the unselected events, and subtracted this value form the sum of the sequences analyzed (11.6 Mb, the yeast genome represented on the microarray [25] x the number of isolates examined). Based on the number of specific genetic elements in the yeast genome, we then calculated the expected number of elements within and outside of the association windows (Columns 4 and 5). These numbers were compared to the observed number of elements within and outside of the association windows by chi-square analysis (Columns 6 and 7).

**Table 1:** Associations between recombination breakpoints and chromosome elements^1^.

Chromosome element^2^	# of elements/genome^2^	Total # in 43 genomes^3^	Expected # inside windows^4^	Expected # outside windows^4^	Observed # inside windows^5^	Observed # outside windows^5^	p values^6^
tRNA genes	275	11782	82	11700	77	11705	0.61
ARS elements	337	13244	92	13152	69	13175	0.019
snRNA and snoRNA genes	83	3569	25	3544	25	3544	1
ncRNA genes	14	602	4	598	6	596	0.45
Ty elements	50	2064	14	2050	12	2052	0.69
Solo long-terminal repeats	231	9460	66	9394	79	9381	0.12
Centromeres	16	688	5	683	3	685	0.5
Palindromic sequences	611	24553	170	24383	159	24394	0.42
G4 sequences (motif 1)	636	23392	162	23230	139	23253	0.08
Highly-transcribed genes	330	14190	99	14091	86	14104	0.21
Weakly-transcribed genes	332	13416	93	13323	118	13298	0.01
Binding sites for Rrm3p	115	4816	34	4782	37	4779	0.67
Regions with high levels of gamma-H2AX	697	27262	189	27073	234	27028	**0.0012**
Replication-termination sequences	71	3053	21	3032	25	3028	0.45
G4 sequences (motif 2)	38	1075	7	1068	6	1069	0.84
G4 sequences (motif 3)	153	3741	26	3715	21	3720	0.37
Regions of high GC content	164	6235	43	6192	50	6185	0.32
Ume6p binding sites	72	3096	21	3075	17	3079	0.45
Sum1p binding sites	37	1591	11	1580	9	1582	0.65

^1^Other details of this analysis are presented in the text and in the S5 Table.

^2^The definitions of each element, their locations, and their numbers in the genome are discussed in the text and in Dataset S8 of Song *et al*. [25].

^3^We analyzed a total of 43 genomes, included both sectored colonies and sub-cloned strains. The number of elements examined with the microarrays is slightly smaller than the number of elements in the genome, since the microarrays do not include sub-telomeric regions [25].

^4^The numbers of genetic elements expected inside and outside of the association windows were calculated as described in the text.

^5^ The numbers of genetic elements observed inside and outside of the association windows were calculated as described in the text.

^6^This column shows p values calculated by chi-square using the observed and expected numbers of events given in Columns 4–7. After correction for performing multiple comparisons, the only p value that was significant was for regions with high levels of gamma-H2AX (shown in boldface).

The unselected events in the sectored colonies and sub-cultured isolates are analyzed and presented both separately and as a composite of both sets of data in S5 Table. We examined associations with 19 genomic elements including tRNA genes, ARS elements, snRNA/snoRNA genes, ncRNA genes, Ty elements, solo long-terminal repeats, centromeres, palindromic sequences, quadruplex (G4) motifs of three different types, highly-transcribed and weakly-transcribed genes, binding sites for the Rrm3p helicase, regions with high levels of the DNA damage-induced H2A modification gamma−H2AX, replication-termination (TER) sites, regions with high GC content, and binding sites for Ume6p and Sum1p (proteins often bound to weakly-transcribed genes). These elements are in the leftmost column of Table 1 (Column 1). The references for the numbers and locations of 17 of these elements in the yeast genome are in Dataset S8 of Song *et al*. [25]. The locations of the Ume6p and Sum1p binding sites are in Szilard *et al*. [13]. The p values for these associations are in Column 8 of Table 1 and in the S5 Table. After correction for multiple comparisons [27], only one association in the composite data was statistically significant. The LOH breakpoints were significantly associated (p = 0.0012) with regions that had high levels of gamma-H2AX [13]. Our interpretation of this association will be discussed below.

## Discussion

From our study of recombination events induced by the hyper-Rec *rad3-102* and *rad3-101* mutations, we conclude: 1) in agreement with previous studies examining gene conversion at specific loci or recombination between repeated sequences [7,24,28,29], our genome-wide analysis shows that these *rad3* mutations stimulate both crossovers and gene conversions by one to two orders of magnitude throughout the genome, 2) the patterns of gene conversions associated with the mitotic crossovers between homologs in *rad3* strains indicate that most of these events are initiated by DSBs occurring in both chromatids, similar to our previous studies of spontaneous events in wild-type cells (14,19), and 3) although most of the recombination events are distributed throughout the genome with no strong hotspots, there is a significant association between LOH breakpoints and regions with high levels of gamma-H2AX.

The simplest interpretation of the hyper-Rec phenotype of the *rad3-102* and *rad3-101* mutations is that these strains have elevated levels of recombinogenic DNA lesions. An alternative possibility is that these strains have the same level of recombinogenic lesions, but the repair of lesions is directed toward recombination between homologs rather than recombination between sister chromatids. Since sister chromatid recombination does not lead to LOH, this type of exchange cannot be detected with our assay. This alternative, however, can be excluded by several arguments. First, using a different assay, Moriel-Carretero and Aguilera [9] showed that *rad-102* and *rad3-101* strains had elevated sister-chromatid recombination, arguing that the frequency of DNA lesions is higher in the mutant strains. Second, Montelone *et al*. [29] found that genomic DNA derived from *rad3-102* strains, analyzed by sucrose gradient centrifugation, was smaller than DNA from wild-type strains, indicating an elevated level of DSBs. Third, Moriel-Carretero and Aguilera [9] showed that *rad3-102* strains have elevated levels of phosphorylated H2A (gamma-H2AX) and Rad52 foci compared to wild-type strains.

The Rad3 proteins encoded by *rad3-102* and *rad3-101* are not defective in helicase function, but fail to complete the repair event, possibly because a prolonged attachment of TFIIH precludes filling-in the gap left by removal of the oligonucleotide with the damaged base [9,10]. Moriel-Carretero and Aguilera [9] proposed that the replication of the nicked chromosome leads to a DSB, and subsequent repair of the DSB by homologous recombination results in a delay of fork progression. In support of this model, they used two-dimensional gel electrophoresis to show an elevated frequency of aberrant replication structures in the *rad3-102* strain. If all DSBs formed in the *rad3* hyper-Rec mutants are a consequence of this mechanism, we would expect that most of the crossovers in these mutants would be associated with 3:1 conversion tracts (Fig 3A). We found, however, that about three-quarters of the conversion tracts had the patterns expected for the repair of two sister-chromatids broken at the same position. Considering both this observation and previous studies, we conclude that two types of DNA lesions occur in the hyper-Rec *rad3* mutants. Most lesions are generated by replicating nicked/gapped DNA molecules, and the resulting single broken chromatids are repaired by sister-chromatid recombination [9], an event that does not result in LOH. However, a second type of lesion also occurs in *rad3* cells, resulting in the breakage of both sister chromatids; this type of event leads to the majority of the recombination events between homologs.

**Fig 7:**
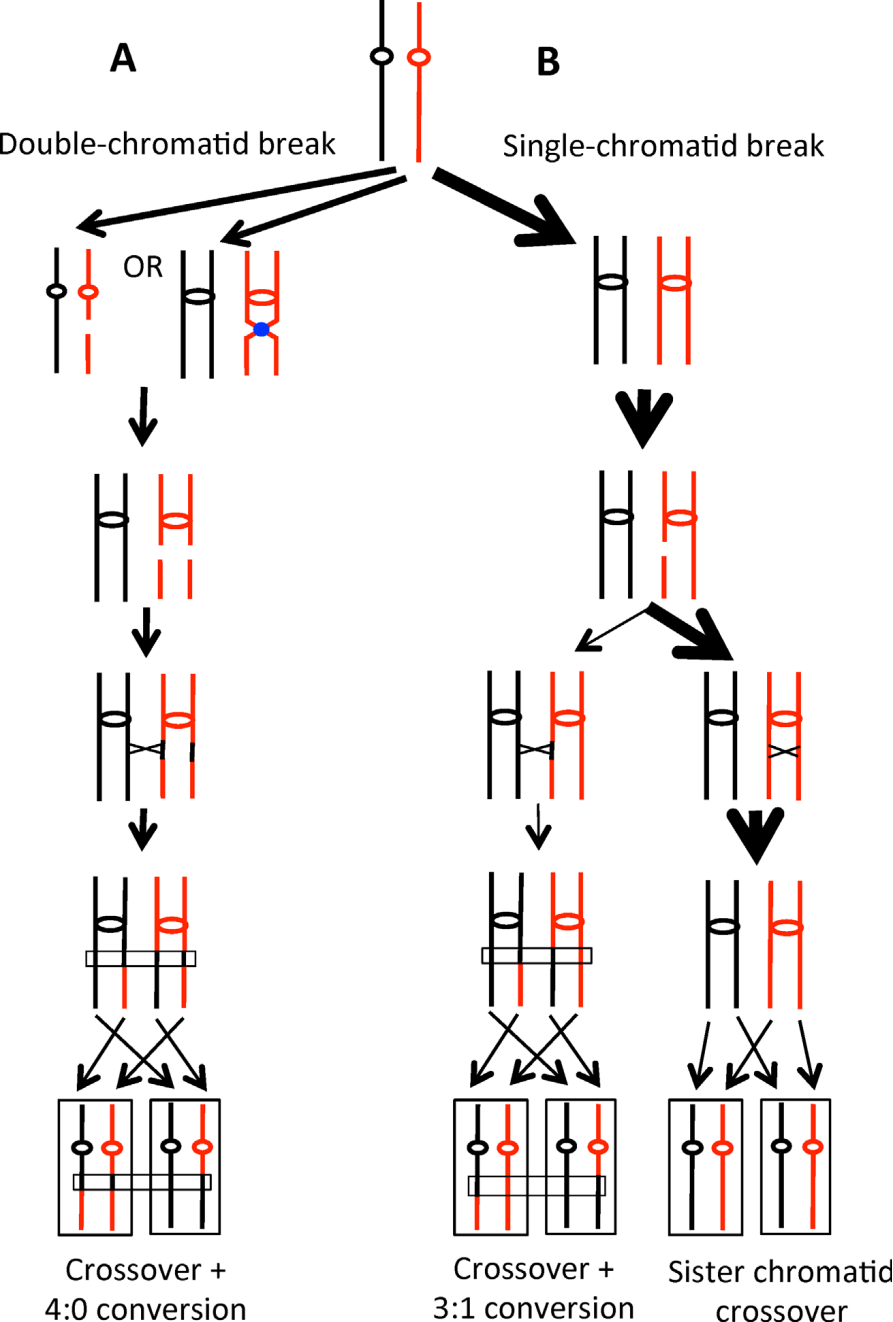
Patterns of repair of single-chromatid or double-chromatid DSBs. Chromosomes are shown as black or red lines, with centromeres indicated by red or black circles or ovals. A TFIIH complex complex is shown as a blue circle. Recombination is initiated by a DSB on the red chromosome/chromatid. The widths of the arrows represents the relative numbers of each class of event. A. Double-chromatid DSBs. On the left side of Fig 7A, the red chromosome broken in G_1_/G_0_ is replicated to yield a pair of sister chromatids broken at the same position. Repair of both chromatids is associated with gene conversion, and one of the repair events is associated with a crossover (indicated by the X). Following the segregation pattern indicated by the dotted arrows, there would be an LOH event with a 4:0 conversion. On the right side of Fig 7A, replication forks converge onto a TFIIH-bound DNA lesion, and breaks occur at the same position on both sister chromatids. B. Single-chromatid DSB. A red chromatid broken during or after DNA replication could be repaired either by recombination with a chromatid of the other homolog (left side of Fig 7B) or with the sister chromatid (right side). The repair event utilizing the chromatid from the other homolog would yield a crossover associated with a 3:1 conversion. Repair involving the sister chromatid would not result in LOH.

What types of DNA damage could produce two sister chromatids that are broken at the same position? We previously showed that DSBs introduced by gamma rays in G_1_-arrested cells produced 4:0 conversion events, whereas DSBs introduced in G_2_-arrested cells primarily result in 3:1 events [21]. We suggested that many spontaneous crossovers between homologs are initiated by DSB formation in G_1_/G_0_ cells followed by replication of the broken chromosome to produce two sister chromatids with breaks at the same position (left half of Fig 7A). Since the two sister chromatids are broken at the same position, use of the sister chromatid as repair substrate is precluded, and the broken chromatids are repaired using the intact homolog [19]. Using a completely different genetic approach, Golin and Esposito [30] argued that some of the recombination events observed in a *rad3-101* strain were a consequence of repair of G_1_-associated DNA lesions. It should be emphasized that the majority of DSBs in the hyper-Rec *rad3* strains are likely to occur in S/G_2_ as shown by the differing widths of the arrows in Fig 7. However, single-chromatid breaks are preferentially repaired by sister-chromatid recombination events [31] that are undetectable by our system.

What mechanism would generate a DSB in G_1_? One obvious possibility is that the DSB results from two very-closely spaced nicks (< 10 bp apart) on opposite strands of the duplex. These nicks could be a consequence of two incomplete NER events or the result of nicks created by two different mechanisms, one related to incomplete NER and the other reflecting a mechanism such as base excision repair (BER) or removal of misincorporated ribonucleotides; approximately 10,000 ribonucleotides are inserted into the yeast genome in each round of replication [32]. Expansion of nicks into gaps mediated by Exo1p or other endonucleases could also affect the frequency of DSBs; loss of Exo1p significantly reduced the levels of G_1_-like recombination events induced by UV treatment [22]. Finally, we point out that the DNA lesion recognized by Rad3p in unirradiated cells is unclear. In addition to cyclopyrimidine dimers, NER proteins are involved in the removal of various types of helix-distorting adducts such as cyclopurines (induced by oxidation) and cis-platin [33].

An alternative possibility is that DSCBs are formed during DNA replication (right side of Fig 7A). From previous studies, it is clear that *rad3-101* and *rad3-102* strains have very high levels of DSBs in S/G_2_, and that these breaks lead to substantially elevated rates of sister-chromatid recombination [9]. It is possible that DNA replication forks converging on a TFIIH-bound DNA lesion may occasionally be processed in a way that results in double sister-chromatid breaks. Similar to the G_1_-associated breaks discussed above, such breaks would have to be repaired by recombination with the intact homolog, leading to LOH.

The conversion tracts associated with crossovers in the *rad3* strains were both longer and more complex than those found in wild-type cells. Although the interpretation of this result is not clear, it is possible that the *rad3* hyper-Rec strains have defects that affect the processing of broken chromosomes, the “patchiness” of mismatch repair, or the tendency to switch templates during repair. It has been previously noted that the hyper-Rec *rad3* mutations in the ATP-binding groove lead to an enhanced attachment of TFIIH to promoter that could challenge DNA repair [10]. Despite these differences in conversion tracts, some features of mitotic recombination in the *rad3-101* and *rad3-102* strains are very similar to spontaneous events in a wild-type strain. In particular, the percentages of events that reflect breakage of a single chromatid or two chromatids were 23% and 77% in the hyper-Rec *rad3* mutants, and 37% and 63% in the wild-type strain [19]. This similarity raises the possibility that some of the DNA lesions that initiate spontaneous recombination are, as established for *rad3* strains, a consequence of incomplete NER events.

The LOH events associated with the *rad3* mutations are distributed throughout the genome (Fig 6). We found no significant association of LOH regions with genomic elements previously shown to be connected with replication-fork barriers such as long terminal repeats (LTRs), binding sites for Rrm3p, and TER sequences. In a study of LOH events induced by low levels of DNA polymerase alpha [25], we observed a strong correlation between LOH breakpoints, and all three of the replication-fork barriers mentioned above.

We did, however, find a significant association between *rad3* LOH breakpoints and regions associated with high levels of gamma-H2AX as mapped by Szilard *et al*. [13]. Phosphorylation of H2A is associated with stalled replication forks and at DSBs [34]. Phosphorylation in response to stalled forks is dependent on Mec1p whereas phosphorylation in response to DSBs is dependent on both Tel1p and Mec1p. In global mapping of regions with high levels of gamma-H2AX in wild-type strains (gamma sites), Szilard *et al*. [13] found that these sites were dependent on both Mec1p and Tel1p, arguing that these sites had elevated levels of DSBs (fragile sites). Gamma sites were enriched for telomeric regions, replication origins, tRNA genes, long-terminal repeats, ribosomal RNA genes, and weakly-expressed genes. Szilard *et al*. also found a correlation between gamma sites and Sum1p and Ume6p binding sites; since these proteins recruit histone deacetylases, this result supports the connection between weakly-expressed genes and gamma sites. In our studies, we observed no connections of LOH breakpoints with weakly-transcribed genes or with binding sites for Sum1p and Ume6p (S5 Table). In summary, our observations suggest that only a sub-set of the fragile sites identified by Szilard *et al*. [13] are associated with elevated recombination between homologous chromosomes. This sub-set may represent regions at which aborted NER events occur even in wild-type cells.

Interestingly, in the present study, we found four events that resulted in deletion or duplication of a sub-telomeric region with breakpoints located near SGD coordinate 25,000 on chromosome XV. Five deletion/duplication events were observed at the same position in strains with low levels of DNA polymerase alpha [25]. As discussed in the Results section, it is likely that these deletions/duplications are a consequence of crossovers or BIR events involving the genes *IMA2* and/or *HXT11* with very similar genes located in the telomeric regions of non-homologous chromosomes. Thus, the *IMA* and/or the *HXT* gene families may represent sequences that are prone to breakage under conditions of replicational and transcriptional stresses.

In summary, our data indicate that there are two types of recombinogenic DNA lesions in strains with the hyper-Rec *rad3* mutations: 1) unrepaired single-stranded nicks that are converted into single chromatid breaks by DNA replication [9] and 2) DSBs or TFIIH-bound DNA lesions in unreplicated chromosomes that are converted into double sister-chromatid breaks by DNA replication. Both types of lesions are repaired during S or G_2_. One of the major unanswered issues is what DNA modification (presumably, a modified base) is recognized by the NER system to initiate formation of DNA nicks and breaks.

## Materials and Methods

### Strain constructions

The strains used in our analysis were derived from two sequence-diverged haploids, W303-1A and YJM789 [14]. The W303-1A-related haploid PG318.1 was constructed by sporulating a diploid formed by crossing RCY317-9a [11] and PSL2 [14]. The genotype of PG318 is: *MATa ade2-1 can1-100 ura3-1 trp1-1 leu2-3*,*113 his3-11*,*15 RAD5 V261553*::*LEU2*. A derivative of PG318 with the *rad3-102* gene and a linked hygromycin-resistant (*hph*) marker replacing the wild-type *RAD3* gene (PGMM1.5) was constructed as described previously [9]. The genotype of the YJM789-derived haploid MD416 [35] is: *MATαade2-1 URA3 can1Δ*::*SUP4-o gal2 ho*::*hisG*. Two isogenic *rad3-102* Hyg^R^ derivatives of MD416 (MDMM.1 and MDMM.9) were constructed by the same approach as used for PGMM1.5. The MDMM.1 and MDMM.9 strains were crossed to PGMM1.5 to produce two isogenic strains of *rad3-102* diploids, MD555 and MD556, respectively. MD416 and PG318.1 were also transformed with *rad3-101* marked with *hph* to make PGMM2.19 and MDMM.30. PGMM2.19 and MDMM.30 were crossed to produce the *rad3-101* diploid used in this work: SLA64.1. Transformants were confirmed with PCR, Southern blots, and UV-sensitivity testing. In addition, the transformants were crossed to strains containing *rad52* and *cdc44-8* mutant strains, and the resulting diploids were dissected to confirm the expected patterns of synthetic lethality.

### Isolation of red/white sectored colonies

Diploid strains were streaked to single colonies on solid rich growth medium (YPD, Yeast Peptone Dextrose) and grown at 30°C for two days. Individual colonies were then diluted in water and plated onto omission plates lacking arginine and containing 10 μg/ml adenine. Plates were incubated at room temperature for three days, followed by an overnight incubation at 4°C overnight. The incubation at 4°C enhances the difference in color between the red and white sectors. To form a red/white sectored colony, cells must undergo a crossover at the time of plating; in colonies in which a crossover occurred after the first division, red, white, and pink sectors are observed within a single colony. The frequency of colonies with red/white sectors (number of sectored colonies/total number of cells in the culture) is, therefore, equivalent to the rate of sector formation per cell division. Because only half of the segregation events following a crossover result in loss of heterozygosity [17], the rate of crossovers is twice the rate of formation of red/white sectored colonies [11].

### Analysis of LOH event using SNP microarrays

Genomic DNA was isolated from cells using the agarose-plug method described by St. Charles *et al*. [12]. The DNA was sonicated to produce fragments of 200–300 bp. DNA from the *rad3* strains was then labeled with Cy5-dUTP, and DNA from a wild-type heterozygous control strain (PG311; [14] or JSC23; [26]) was labeled with Cy3-dUTP using the Invitrogen Bioprime Array CGH Genome Labeling Kit. The labeled samples were mixed and hybridized to Agilent oligonucleotide SNP microarrays [12,19]. The arrays were scanned at wavelengths of 635 and 532 nm using the GenePix Scanner and GenePixPro software. The data were then exported to text files and analyzed with Microsoft Excel. We determined the ratio of the median hybridization values (635 nm/532 nm) for all probes individually. Following several steps of normalization [12], heterozygous SNPs had a ratio of about one. Genomic regions homozygous for the W303-1A-specific alleles hybridized with a ratio of about 1.5 to W303-1A-specific oligonucleotides, and with a ratio of about 0.2–0.3 to YJM789-specific oligonucleotides; regions homozygous for YJM789-specific alleles had the reciprocal ratios. The design of the microarrays (whole-genome and chromosome V-specific) and all data from the 96 microarrays used in our analysis are available on the NCBI Gene Expression Omnibus (GEO) database (GSE76395).

### Association of LOH events and genomic elements

Using the procedures described in the text and in Song *et al*. [25], we determined the regions associated with the unselected LOH events that were likely to contain the site of the recombinogenic windows. We then determined whether these association windows had an over-representation of various types of genomic elements. Nineteen different elements were examined. The list of these elements is in S5 Table. In this table, we also summarize our analysis for unselected events in sectored colonies and in sub-cultured *rad3* strains.

### Statistical tests

For various features of the data, we used the chi-square test, the Fisher exact test, or the non-parametric Mann-Whitney test. These analyses were performed using the VassarStats Website (http://www.vassarstats.net). To correct p values when multiple tests were performed, we used the procedure of Hochberg and Benjamini [27].

## Supporting Information

S1 Table Sequences of oligonucleotides used for SNP analysis on the left arm of chromosome V. (XLSX)

S2 Table Classes of LOH events in sectored colonies. (XLS)

S3 Table Location of LOH events on chromosome V. (XLSX)

S4 Table Location of unselected LOH events throughout the genome. (XLSX)

S5 Table Correlation of breakpoints of LOH events with genomic elements. (XLSX)
